# Supplementary material for: Effects of aircraft noise exposure on self-reported health through aircraft noise annoyance: Causal mediation analysis in the DEBATS longitudinal study in France
Source: PLoS One. 2024 Aug 27;19(8):e0307760. doi: 10.1371/journal.pone.0307760 (PMC11349086; doi:10.1371/journal.pone.0307760)
Supplement: S1 Table — (DOCX) [file pone.0307760.s001.docx]

**S1 Table:** **Causal interventional effects of aircraft noise levels in terms of odds ratio (OR) for poor/fair self-reported health, sensitivity analyses**

| **Using marginal M3 and M4 models** | | | | | | |
| --- | --- | --- | --- | --- | --- | --- |
|  | **50-54 vs < 50 dBA** |  | **55-59 vs < 50 dBA** |  | **≥ 60 vs < 50 dBA** | |
|  | OR (95%CI^1^) |  | OR (95%CI^1^) |  | OR (95%CI^1^) | |
| Total interventional effect | 1.06 (0.89 to 1.42) |  | 1.13 (0.82 to 1.97) |  | 1.19 (0.78 to 2.61) | |
| Direct interventional effect | 1.02 (0.83 to 1.36) |  | 1.04 (0.72 to 1.75) |  | 1.07 (0.65 to 2.23) | |
| Indirect interventional effect | 1.04 (0.96 to 1.17) |  | 1.08 (0.92 to 1.36) |  | 1.12 (0.89 to 1.59) | |
| Mediated proportion^2^ | 67% |  | 63% |  | | 65% |
| **Using M1 and M2 models and aircraft noise annoyance as a binary variable** | | | | | | |
| Total interventional effect | 1.07 (0.89 to 1.44) |  | 1.17 (0.87 to 2.06) |  | | 1.33 (0.94 to 3.17) |
| Direct interventional effect | 1.03 (0.82 to 1.37) |  | 1.06 (0.70 to1.80) |  | | 1.09 (0.63 to 2.23) |
| Indirect interventional effect | 1.04 (0.99 to 1.14) |  | 1.10 (0.99 to 1.47) |  | | 1.22 (1.00 to 2.15) |
| Mediated proportion^2^ | 58% |  | 61% |  | | 70% |
| **Using M1 and M2 models and truncated weights** | | | | | | |
| Total interventional effect | 1.07 (0.93 to 1.40) |  | 1.15 (0.86 to 1.95) |  | | 1.23 (0.77 to 2.59) |
| Direct interventional effect | 1.03 (0.85 to 1.33) |  | 1.06 (0.73 to 1.76) |  | | 1.09 (0.62 to 2.28) |
| Indirect interventional effect | 1.04 (0.96 to 1.14) |  | 1.09 (0.93 to 1.31) |  | | 1.13 (0.87 to 1.48) |
| Mediated proportion^2^ | 58% |  | 62% |  | | 59% |
| **Using M1 and M2 models with additional control for pre-existing diseases^3^** | | | | | | |
| Total interventional effect | 1.06 (0.88 to 1.46) |  | 1.13 (0.99 to 1.64) |  | | 1.20 (0.99 to 2.10) |
| Direct interventional effect | 1.01 (0.82 to 1.38) |  | 1.03 (0.91 to 1.40) |  | | 1.04 (0.86 to 1.67) |
| Indirect interventional effect | 1.05 (0.96 to 1.22) |  | 1.10 (1.03 to 1.24) |  | | 1.15 (1.05 to 1.39) |
| Mediated proportion^2^ | 83% |  | 77% |  | | 76% |
| ^1^ Confidence intervals obtained with a bootstrap of 7,500 resamples (convergence rate: 3,804/7,500) using the bootstrap bias  correction method.  ^2^Mediated proportion $= {\log\left( \mathrm{OR}_{\mathrm{IIE}} \right)}/{\log\left( \mathrm{OR}_{\mathrm{TIE}} \right)}$ where IIE corresponds to indirect interventional effect and TIE to total  interventional effect.  ^3^This variable corresponded to the presence of at least one comorbidity diagnosed in the last 12 months prior to the interview, from among the following: diabetes, cardiovascular diseases, myocardial infarction, cancer, diagnosed hypertension and medication use. | | | | | | |
