# Supplementary material for: Effects of aircraft noise exposure on self-reported health through aircraft noise annoyance: Causal mediation analysis in the DEBATS longitudinal study in France
Source: PLoS One. 2024 Aug 27;19(8):e0307760. doi: 10.1371/journal.pone.0307760 (PMC11349086; doi:10.1371/journal.pone.0307760)
Supplement: S1 Fig — (DOCX) [file pone.0307760.s002.docx]

**A**


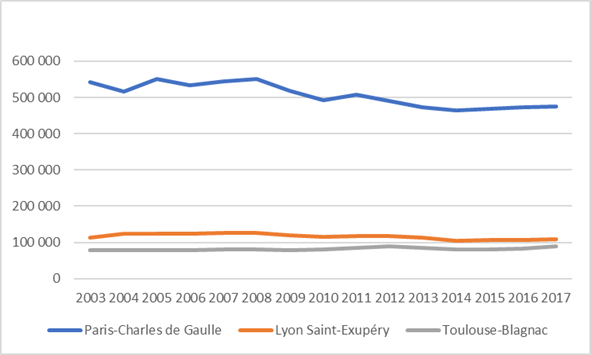


**B**


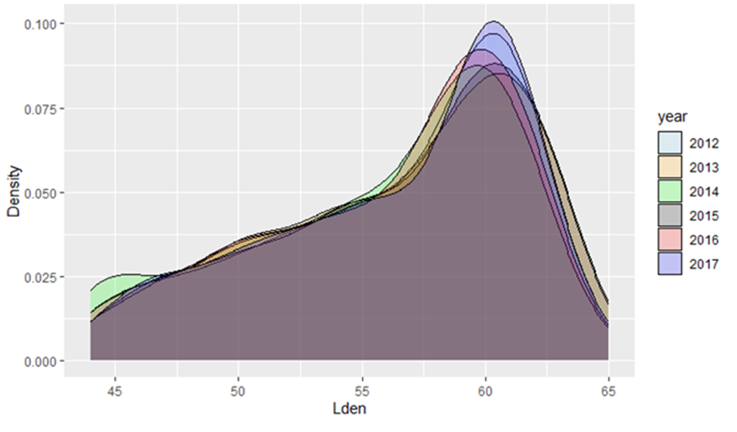


**S1 Fig. Evolution of the number of movements and aircraft noise levels distribution.** (A) number of movements from 2003 to 2017 for the three airports in the DEBATS study. (B) distribution of aircraft noise levels for Paris-Charles de Gaulle airport for participants who don't move from 2012 to 2017.
