# Supplementary material for: Effects of aircraft noise exposure on self-reported health through aircraft noise annoyance: Causal mediation analysis in the DEBATS longitudinal study in France
Source: PLoS One. 2024 Aug 27;19(8):e0307760. doi: 10.1371/journal.pone.0307760 (PMC11349086; doi:10.1371/journal.pone.0307760)
Supplement: Attachment — Submitted filename: Response to Reviewers.docx [file pone.0307760.s003.docx]

Response to Reviewers

The co-authors would like to thank the reviewers for their comments, which give them the opportunity to clarify certain aspects.

The lines indicated in the responses to the reviewers correspond to the lines in the revised versions of the manuscript with track changes.

Journal requirements:

When submitting your revision, we need you to address these additional requirements.

1. Please ensure that your manuscript meets PLOS ONE's style requirements, including those for file naming. The PLOS ONE style templates can be found at

https://journals.plos.org/plosone/s/file?id=wjVg/PLOSOne_formatting_sample_main_body.pdf and

https://journals.plos.org/plosone/s/file?id=ba62/PLOSOne_formatting_sample_title_authors_affiliations.pdf

2. Please note that funding information should not appear in any section or other areas of your manuscript. We will only publish funding information present in the Funding Statement section of the online submission form. Please remove any funding-related text from the manuscript.

The funding-related text has been removed from the manuscript.

3. Thank you for stating the following financial disclosure:

"The present study was supported by funds from the French Ministry of Health, the French Ministry of the Environment, the French Civil Aviation Authority, the Airport Pollution Control Authority (Acnusa) and the Gustave Eiffel University. The authors would like to thank them for their kind assistance."

Please state what role the funders took in the study. If the funders had no role, please state: "The funders had no role in study design, data collection and analysis, decision to publish, or preparation of the manuscript."

If this statement is not correct you must amend it as needed.

Please include this amended Role of Funder statement in your cover letter; we will change the online submission form on your behalf.

"The funders had no role in study design, data collection and analysis, decision to publish, or preparation of the manuscript." This information has been included in the cover letter.

4. For studies involving third-party data, we encourage authors to share any data specific to their analyses that they can legally distribute. PLOS recognizes, however, that authors may be using third-party data they do not have the rights to share. When third-party data cannot be publicly shared, authors must provide all information necessary for interested researchers to apply to gain access to the data. (https://journals.plos.org/plosone/s/data-availability#loc-acceptable-data-access-restrictions)

For any third-party data that the authors cannot legally distribute, they should include the following information in their Data Availability Statement upon submission:

1) A description of the data set and the third-party source

2) If applicable, verification of permission to use the data set

3) Confirmation of whether the authors received any special privileges in accessing the data that other researchers would not have

4) All necessary contact information others would need to apply to gain access to the data

The data that support the findings of this study are not publicly available because they are covered by national agreements concerning the ethical use of data and the protection of confidentiality of individuals in France. Data are however available from the authors upon reasonable request and with permission of the French authorities. Please contact the corresponding author with permission from these authorities.

5. Your ethics statement should only appear in the Methods section of your manuscript. If your ethics statement is written in any section besides the Methods, please move it to the Methods section and delete it from any other section. Please ensure that your ethics statement is included in your manuscript, as the ethics statement entered into the online submission form will not be published alongside your manuscript.

The ethics statement has been moved to the Methods section.

6. Please include captions for your Supporting Information files at the end of your manuscript, and update any in-text citations to match accordingly. Please see our Supporting Information guidelines for more information: http://journals.plos.org/plosone/s/supporting-information.

[Note: HTML markup is below. Please do not edit.]

Reviewers' comments:

Reviewer's Responses to Questions

Comments to the Author

1. Is the manuscript technically sound, and do the data support the conclusions?

The manuscript must describe a technically sound piece of scientific research with data that supports the conclusions. Experiments must have been conducted rigorously, with appropriate controls, replication, and sample sizes. The conclusions must be drawn appropriately based on the data presented.

Reviewer #1: Partly

Reviewer #2: Yes

Reviewer #3: Partly

2. Has the statistical analysis been performed appropriately and rigorously?

Reviewer #1: Yes

Reviewer #2: Yes

Reviewer #3: Yes

3. Have the authors made all data underlying the findings in their manuscript fully available?

The PLOS Data policy requires authors to make all data underlying the findings described in their manuscript fully available without restriction, with rare exception (please refer to the Data Availability Statement in the manuscript PDF file). The data should be provided as part of the manuscript or its supporting information, or deposited to a public repository. For example, in addition to summary statistics, the data points behind means, medians and variance measures should be available. If there are restrictions on publicly sharing data—e.g. participant privacy or use of data from a third party—those must be specified.

Reviewer #1: Yes

Reviewer #2: Yes

Reviewer #3: Yes

4. Is the manuscript presented in an intelligible fashion and written in standard English?

PLOS ONE does not copyedit accepted manuscripts, so the language in submitted articles must be clear, correct, and unambiguous. Any typographical or grammatical errors should be corrected at revision, so please note any specific errors here.

Reviewer #1: Yes

Reviewer #2: Yes

Reviewer #3: Yes

5. Review Comments to the Author

Please use the space provided to explain your answers to the questions above. You may also include additional comments for the author, including concerns about dual publication, research ethics, or publication ethics. (Please upload your review as an attachment if it exceeds 20,000 characters)

**Reviewer #1:**

Summary: Based on longitudinal data of three waves with interviews between 2013 and 2017 of 881 participants, causal pathways from aircraft noise exposure to self-reported health status are studied using marginal structural models with inverse probability weighting. It was found that the indirect effect through noise annoyance was dominant over the direct pathway through noise exposure.

Main impression: The DEBATS study from France has already produced several interesting publications with important insights in the effect chains from aircraft noise exposure to detrimental health effects. The current analysis shows a methodically interesting design and contributes to the discussion about the importance of indirect pathways.

Q1- Personally, I see one fundamental weakness of the study, namely the fact that the exposure data of two of the three airports was not corresponding to the survey years and in addition that it was assumed to be constant over all three years. With aircraft noise exposure being one of the two core input parameters for the modelling, the data quality seems not good enough for a longitudinal study and to draw these conclusions. The results of the structural equations models as given in Figure 2 support my concern as the association between Lden and SRHS changes sign from year to year, which seems rather unlikely to me.

Therefore, I suggest to refine the exposure modelling or at least to justify the assumption that the aircraft noise exposure of the years 2013, 2015 and 2017 was identical to 2003/2004. In order to do that, the following steps should be taken: (a) compare the number of movements, ideally separately for day, evening and night, (b) insure that the fleet mix remained stable (no introduction of new aircraft types) and (c) that the distribution of routes remained stable. This comparison should also be done for Paris-Charles de Gaulle airport and the outcome should be compared with the available aircraft noise contours of Paris-Charles de Gaulle.

In parallel, it would be interesting to rerun the models using exclusively the data of Paris, which exhibit better quality input data.

We had access only to the numbers of annual movements in Lyon Saint-Exupéry and Toulouse-Blagnac airports in 2013, 2015 and 2017, which do not differ to the one observed in 2003/2004. This information is not available for daytime, evening and night-time, separately. Figure 1 below shows trends in the number of movements between 2003 and 2017 for the three airports. Over time, the number of movements tends to decrease slightly at Paris-Charles de Gaulle airport, while no evolution was observed at Lyon Saint-Exupéry and Toulouse-Blagnac airports.

Figure 1: Number of movements between 2003 and 2017 for the three airports ([1])

For Paris-Charles de Gaulle airport, noise maps were available to calculate aircraft noise levels between 2012 and 2017. Figure 2 below shows the distribution of these aircraft noise levels for participants in the DEBATS study who had not moved between 2012 and 2017. It should be noted that it has remained stable over time.

Figure 2: Distribution of aircraft noise levels for participants near Paris-Charles de Gaulle airport who had not moved between 2012 and 2017

In another study, Bruitparif, the noise observatory for the Ile-de-France region, showed that for Paris-Charles de Gaulle airport, changes in traffic alone lead to relatively small variations of around +/- 0.4 dB [2].

Thus, we assumed that the aircraft noise exposure of the years 2013, 2015 and 2017 does not differ from that observed in 2003/2004. Potential changes in aircraft noise levels are very likely to be lower than 5 dBA, which corresponds to the range of noise classes within which participants are grouped in this study. A classification bias when assessing the noise class cannot be ruled out, but this potential bias is likely non-differential since unrelated to the SRHS outcome and would lead in this study to an underestimation of the real effects of aircraft noise levels.

The following paragraph has been included in the Discussion section, lines 391-402: “Indeed, the number of aircraft movements did not change over time for the three airports included in the DEBATS study (Fig A in S1 Fig). In addition, using the noise maps available for Paris-Charles de Gaulle airport between 2012 and 2017, the exposures to aircraft noise levels of participants who had not moved during this period did not differ to the one observed in 2013 (Fig B in S1 Fig). In another study, Bruitparif, the noise observatory for the Ile-de-France region, showed that for Paris-Charles de Gaulle airport, changes in traffic alone lead to relatively small variations of around +/- 0.4 dB [32]. Potential changes in aircraft noise levels would be very likely lower than 5 dBA, which corresponds to the range of noise classes within which participants are grouped in this study. A classification bias when assessing the aircraft noise level cannot be ruled out, but this potential bias is likely non-differential since unrelated to the SRHS outcome and would lead in this study to an underestimation of the real effects of aircraft noise levels.”

Table 1 below shows the description of aircraft noise levels and annoyance according to the participants’ self-reported health status for participants, four the three visits, around Paris-Charles de Gaulle airport only. A total of 369 participants were included, 62 of whom reported fair/poor SRHS at T0, 60 at T1, and 55 at T2. Consequently, the number of participants reporting fair/poor SRHS in the different categories of aircraft noise was very low, particularly for the <50 dBA and 50-55 dBA categories. Indeed, in the DEBATS study, stratification was conducted to ensure a sufficient number of participants in each noise level category. Thus, participants in the <50 and 50-55 categories were mainly residents around Lyon-Saint-Exupery and Toulouse-Blagnac airports, where noise levels are lower than in Paris-Charles de Gaulle airport. The number of participants reporting fair/poor SRHS and who reported being extremely or very annoyed by aircraft noise was low for the mediation analysis. Thus, as the results of the models using only the Paris-Charles de Gaulle data were not robust, and presented a large variance in the estimates, we chose not to present them in this article.

Table 1: Aircraft noise levels and annoyance according to self-reported health status, for the three visits, for Paris-Charles de Gaulle airport’s participants

|  |  | T0 | | T1 | | T2 | |
| --- | --- | --- | --- | --- | --- | --- | --- |
|  |  | Good/excellent | Fair/poor | Good/excellent | Fair/poor | Good/excellent | Fair/poor |
|  | N | 307 | 62 | 309 | 60 | 314 | 55 |
| L_den_ (in dBA) | |  |  |  |  |  |  |
| <50 | | 42 (14%) | 3 (5%) | 45 (15%) | 7 (12%) | 50 (16%) | 6 (11%) |
| 50-54 | | 70 (23%) | 11 (18%) | 59 (19%) | 15 (25%) | 64 (20%) | 11 (20%) |
| 55-59 | | 85 (28%) | 21 (34%) | 89 (29%) | 19 (32%) | 80 (26%) | 18 (33%) |
| ≥ 60 | | 110 (36%) | 27(44%) | 116 (37%) | 19 (32%) | 119 (38%) | 20 (36%) |
| Aircraft noise annoyance | |  |  |  |  |  |  |
|  | Extremely | 50 (16%) | 9 (15%) | 39 (13%) | 6 (10%) | 57 (18%) | 10 (18%) |
|  | Very | 80 (26%) | 11 (18%) | 74 (24%) | 14 (23%) | 75 (24%) | 8 (15%) |
|  | Moderately | 110 (36%) | 25 (40%) | 113 (37%) | 23 (38%) | 120 (38%) | 20 (36%) |
|  | Slightly | 56 (18%) | 14 (23%) | 66 (21%) | 14 (24%) | 54 (17%) | 14 (25%) |
|  | Not at all | 11 (4%) | 3 (5%) | 17 (5%) | 3 (5%) | 8 (3%) | 3 (6%) |

Comments in detail:

L. 48 This is the first time SRHS is used in the text. Therefore I suggest to use the full expression including "status".

Status has been included.

L. 77 "Tchetgen" comes twice.

It’s not a mistake, it’s really the author’s name.

L. 100ff As discussed above…

This discussion has been included to the Discussion section lines 391-402 (see previous answer to Q1).

L. 132 Please indicate how the quality of sound insulation was determined

The following information has been included in the Methods section, lines 164-167: “Windows or roof insulation was self-reported by participants in response to the question "Does your dwelling have roof (or windows) insulation against external noise?" The dwelling was considered to be insulated if at least one of the roof or windows was insulated.”

L. 154 The sentence ends with a bracket instead of a point. The bracket is closed on line 161.

We have checked: this typo was absent from our original document and occurred when the PDF version was generated automatically.

L. 157 This should be part of the caption, but looks like normal text.

We have checked: this problem was absent from our original document and occurred when the PDF version was generated automatically.

L. 167 The caption of Figure 1 is repeated and should be deleted.

We have checked: this repetition was absent from our original document and occurred when the PDF version was generated automatically.

L. 210 "et" instead of "and"?

This has been corrected.

L. 251 "Aircraft noise levels seems affected SRHS through annoyance." Please revise this sentence.

The sentence has been revised as follows: “Aircraft noise levels seems to affect SRHS through annoyance.”

L. 266 Fig. 3, at least in the way it is given at the end of the document, shows a bad resolution and is hard to read.

We have tried to improve the resolution. If the image resolution remains poor in the automatically generated PDF, a link at the top of the image allows access to the image with the correct resolution.

L. 371 "But this bias seems very unlikely. … showed no significant difference in exposure levels." As previously mentioned, this conclusion should be justified further. How do you assess whether a change in exposure is significant or not?

As mentioned above, this discussion has been added to the Discussion section.

**Reviewer #2:**

This is a very interesting study aimed at determining the causal mediating role of aircraft noise annoyance in the association between aircraft noise levels and self-reported health. The authors are experienced in the field, as they have previously published interesting data from the DEBATS study. This is a valuable contribution to the research field with very novel and robust methodology, as there is an ongoing discussion about the role of noise annoyance. I guess the major weakness of this study is the outcome, as the health status is evaluated by only one item. I have only minor comments:

Q1- Abstract: Could you provide some odds ratios (ORs) so that one can get a sense of the magnitude of effects?

Odds ratios have been included in the abstract.

Q2 -Aircraft noise exposure: Does it make sense to present the results with continuous aircraft noise levels as well?

The following paragraph has been included in the Discussion section, lines 436-443: “Ideally, from a statistical standpoint, considering noise levels as continuous variables seems more favorable to avoid losing information typically due to categorization. However, the IPTW estimation method used in this study requires a good understanding of the distribution of aircraft noise levels [3]. This distribution is often assumed to be a normal distribution. In this study, the distribution diverged from a normal distribution at all (Cf. Figure 3). In the case of unknown distribution, categorization of the variable remains the alternative solution [3]. We have therefore opted for a categorization into four classes corresponding to the different aircraft noise contours of the three airports included in the DEBATS study.”

Figure 3: Aircraft noise levels distribution for the three visits in the DEBATS study

Q3- With the term “gender” I assume you mean “sex”.

Not really. The gender was recorded by the interviewer without asking the participant. It may therefore differ from the biological sex.

Q4- Do you have data on pre-existing diseases or newly developed diseases at follow-up and medication intake?

We have information on the existence of certain pre-existing diseases such as cardiovascular disease, diabetes, cancer, diagnosed hypertension, or the use of antihypertensive medication or of other medication. Some of these have already been the subject of published articles and others will be the subject of future publications.

Q5- Is there any chance to “validate” the outcome with more objective or subjective data?

Validating SRHS using more objective or subjective data is beyond the scope of the results presented in this article and could not be achieved within a reasonable timeframe.

SRHS is widely used to measure the general health of populations. This is a multi-dimensional indicator that considers health and lifestyle practices, functional, coping and well-being dimensions[4, 5]. It has been shown to be a good predictor of quality of life, morbidity and mortality [6, 7]. This definition has been added in the introduction, lines 62-65.

In addition, the following paragraph has been included in the revised manuscript (lines 406-410) (see response to reviewer 3): “SRHS was assessed using a unique subjective question. Response to this question by the participant could be filtered through their perceptions and could thus not reflect their real multidimensional health. In addition, as DEBATS is a study on the health effects of aircraft noise, participants’ responses could be prone to response bias, particularly with regards to self-reporting of SRHS and annoyance.”

Q6- Could you propose what might account for the remaining 35% when 65% of the effect is mediated by noise annoyance? From my understanding, there was no direct effect of aircraft noise on health status. Does this imply that only when annoyance occurs as a response, it influences health status? In other words, would it be correct to state that without annoyance, there is no adverse impact on health?".

Part of the remaining 35% (i.e. not mediated by annoyance) could be explained by other intermediate factors which may also play a mediating role, as we have already indicated: “The mediation analysis presented in this study was limited to considering aircraft noise annoyance as the only mediator. However, factors associated with annoyance, such as sleep disturbance and stress, could mediate the causal effect of aircraft noise levels on health. […] Therefore, a mediation analysis jointly assessing several mediators may provide a better identification of the causal effect of noise levels on health”.

The paragraph has been amended as follows (lines 444-448): “Almost 65% of the effect of aircraft noise on fair/poor SRHS is mediated by noise annoyance. The mediation analysis presented in this study was limited to considering aircraft noise annoyance as the only mediator. Factors associated with annoyance, such as sleep disturbance and stress, could mediate the causal effect of aircraft noise levels on health, and explain part of the remaining 35% of the total effect.”

**Reviewer #3:**

This study uses a longitudinal cohort study in France along with estimates of participants’ aircraft noise exposures to investigate mediation of effects on self-reported health through self-reported annoyance. The design is innovative for its application of a causal mediation framework to help elucidate – how transportation noise, particularly aircraft noise, may impact health, and through which mechanisms. While the design is robust and uniquely applied, there are some important limitations that the authors do not address thoroughly.

Q1- The authors mention that there are unmeasured mediators that are likely to play a role in health and hint at the likelihood for unmeasured confounders. And yet they assume they have correctly specified the model. These two ideas are incongruous. I would suspect that, for the reasons the authors mentioned, the model is indeed misspecified (though no major fault of the authors – the right data is rarely, if ever, actually available, and such efforts are inherently idealistic but laudable, no less). And that is why greater nuance in the reporting of the results needs to be added in key places throughout the manuscript (abstract, discussion, conclusion). Adding in these factors may change the indirect effects of annoyance dramatically. Lines 337-340 are an example of overly strong language that needs to be toned back. This exercise still has great utility and demonstrates a recipe for other researchers to follow, but there are important assumptions in causal inference as there are with other techniques that are not given enough attention, in my opinion.

This comment was considered in the revised manuscript.

Lines 356-359 have been revised as follows: “This result suggests importance of aircraft noise annoyance in the causal effect of exposure to aircraft noise levels on poor/fair SRHS”. And the following sentence has been removed: “Almost all of the deleterious effect of aircraft noise levels on SRHS would be due aircraft noise annoyance”.

The potential misspecification bias has been mentioned by including the following sentence in the lines 409: “Potential model misspecification bias cannot be ruled out”.

It was not possible to evaluate the mediating effect of several mediators in this study, which is one of its limitations, as we have already mentioned in the discussion (lines 444-448):

“The mediation analysis presented in this study was limited to considering aircraft noise annoyance as the only mediator. Factors associated with annoyance, such as sleep disturbance and stress, could mediate the causal effect of aircraft noise levels on health, and explain part of the remaining 35% of the total effect. […].

Q2- One subjective question on SRHS is a coarse and crude metric upon which to base a study. Granted, the design is thoughtful; but, it cannot be overstated that this is a single dimension of health perceived by the participant measured in a highly subjective fashion. Presumably, participants are filtering responses through their perceptions, and many of these are fundamentally mental processes. The lack of objectivity does not allow the researchers to understand the dimensions of health, whether even physical or mental, much less the other subdimensions of health. This major limitation needs to be added to the limitations section, at minimum.

This limitation has been included in the revised manuscript (lines 406-410) as follows: “SRHS was assessed using a unique subjective question. Response to this question by the participant could be filtered through their perceptions and could thus not reflect their real multidimensional health. In addition, as DEBATS is a study on the health effects of aircraft noise, participants’ responses could be prone to response bias, particularly with regards to self-reporting of SRHS and annoyance.”

Q3- Relatedly, I think there is decent potential for reverse causality despite the innovative design that does include the relationship between SRHS and subsequent annoyance. However, I would find it somewhat difficult to believe that SRHS and annoyance are not correlated and intertwined at the same time point, particularly at baseline. The orange arrow in Fig 1 from annoyance to SRHS at baseline is a strong assumption. How can the authors rule out reverse causality here? I could see how perceived SRHS could make increase annoyance. I think this point is major and not minor. The authors partially address this through their time-varying design at later time points; but I am failing to see how the self-reported nature of similar concepts (health and annoyance) are not fundamentally related. If they share a common ancestor from the causal inference perspective, there would be bias introduced by improper/lack of conditioning. I think this point is nuanced but important, and not as well articulated as it should be.

It is indeed conceivable that both aircraft noise annoyance and SRHS being subjective and self-reported at the same time, perceived SRHS could influence the reported annoyance. Participants were asked about their noise annoyance in the 12 months preceding the interview, while they were asked to report their SRHS on the day of the interview. These two variables were thus ordered in favor of the hypothesis that aircraft noise annoyance affects SRHS.

In addition, confounding bias could occur since aircraft noise annoyance and SRHS share a common ancestor from the causal inference perspective. The inverse probability weighting (IPW) approach that mimics interventional setting for controlling for this confounding was used [8].

This point has been included in the revised manuscript (lines 430-435) as follows: “At the same time point, an effect of annoyance on SRHS was assumed (Fig 1) since participants were asked to report their noise annoyance in the 12 months preceding the interview and their SRHS on the day of the interview. A reciprocal effect of SRHS on the reporting of annoyance, inherent to the subjective nature of the exposure and of the outcome, could not be ruled out and could induce misspecification bias. This potential reciprocal effect was partially addressed through a time-varying design at later time points (Fig 1).”

Q4- The DEBATS study is a study on the health effects of aircraft noise. As such, participants are prone to response bias, particularly in the self-reporting of SRHS and annoyance. That should be mentioned as an additional limitation and considered throughout the study.

This limitation has been included in the revised manuscript, lines 408-410 as follows: “as DEBATS is a study on the health effects of aircraft noise, participants’ responses could be prone to response bias, particularly with regards to self-reporting of SRHS and annoyance.

Q5- It’s too bad that 2/3 airports had outdated and non-time varying aircraft noise exposure estimates. Did I miss somewhere the changes in aircraft noise exposures among participants over time at CDG? If not, I think it may be useful to show, at minimum, the changes in exposures for participants living around CDG that did not move over the study period by year, perhaps in the supplement. It would enable crude justification that the patterns at the other 2 airports were similar. The authors could consider a quantitative bias analysis if they thought prudent.

Justification and further information have been included, lines 391-402 in the revised manuscript (see also responses to question 1 for the reviewer 1).

Q6- Why did the author not consider adding analyses of dichotomized aircraft noise exposures at different cut points, and only investigate it as categorical? Is an analysis of continuous exposure possible in this framework?

Ideally, from a statistical standpoint, considering noise levels as continuous variables seems more favorable to avoid losing information typically due to categorization. However, the IPTW estimation method used in this study requires a good understanding of the distribution of aircraft noise levels [3]. This distribution is often assumed to be a normal distribution. In our study, the distribution diverged from a normal distribution (Cf. Figure 3, response to the question 2 of the reviewer 2). In the case of unknown distribution, categorization of the variable remains the alternative solution [3]. We have therefore opted for a categorization into four classes corresponding to the different aircraft noise contours of the three airports included in the DEBATS study.

Table 2 below shows the results obtained using dichotomized aircraft noise exposures from different cut points (50, 55 and 60 dBA). The OR for the total interventional effect across all cut points did not reach statistical significance. Using the 50 dBA cut point, the results suggest a complete mediating effect through aircraft noise annoyance, even if this indirect effect was not significant. In fact, the OR for the indirect effect was equal to the OR for the total effect. However, this mediating effect disappeared in the analysis using the 55 and the 60 dBA cut points. The use of dichotomized aircraft noise levels does not provide sufficiently contrasting exposures to have sufficient statistical power. In addition, this may lead to high loss of information compared to the categorization into four classes, particularly for identification of potential mediation effect of aircraft noise annoyance (for example, when considering the cut points 55 and 60 dBA in this study). Consequently, we do not show these results in the article.

Table 2: Interventional causal effects of aircraft noise levels in terms of odds ratio (OR) for poor/fair self-reported health status using dichotomized aircraft noise levels.

|  | **≥ 50 vs < 50 dBA** | **≥ 55 vs < 55 dBA** | **≥ 60 vs < 60 dBA** |
| --- | --- | --- | --- |
|  | OR (95%CI^1^) | OR (95% CI^1^) | OR (95% CI^1^) |
| Total interventional effect | 1.07 (0.74 to 2.50) | 1.31 (0.98 to 2.30) | 1.18 (0.67 to 2.00) |
| Direct interventional effect | 1.00 (0.68 to 2.18) | 1.30 (0.95 to 2.32) | 1.17 (0.66 to 2.04) |
| Indirect interventional effect | 1.07 (0.91 to 1.37) | 1.01 (0.87 to 1.22) | 1.00 (0.90 to 1.23) |

The same footnote for Fig 1 needs to be included for Fig 2.

This has been done.

Lines 251-252: typo/incomplete sentence

The sentence has been reformulated as follows: “Aircraft noise levels seems to affect SRHS through annoyance.”

Table 2: there should be no acronyms in the title (e.g., SRHS) to help it be “standalone”.

SRHS has been replaced with Self-rated health status.

6. PLOS authors have the option to publish the peer review history of their article (what does this mean?). If published, this will include your full peer review and any attached files.

If you choose “no”, your identity will remain anonymous but your review may still be made public.

Do you want your identity to be public for this peer review? For information about this choice, including consent withdrawal, please see our Privacy Policy.

Reviewer #1: No

Reviewer #2: No

Reviewer #3: No

References

1.	Union des Aéroports Français. https://www.aeroport.fr/view-statistiques/paris-charles-de-gaulle. Accessed 2 May 2024

2.	Bruitparif (2019) Rapport - Résultats des mesures de bruit aérien réalisées en 2018 dans le cadre de l’action SURVOL du PRSE3.

3.	Naimi AI, Moodie EEM, Auger N, Kaufman JS (2014) Constructing Inverse Probability Weights for Continuous Exposures: A Comparison of Methods. Epidemiology 25:292–299

4.	Yamada C, Moriyama K, Takahashi E (2012) Self-rated health as a comprehensive indicator of lifestyle-related health status. Environmental health and preventive medicine 17:457–462

5.	Simon JG, De Boer JB, Joung IM, Bosma H, Mackenbach JP (2005) How is your health in general? A qualitative study on self-assessed health. The European Journal of Public Health 15:200–208

6.	Idler EL, Benyamini Y (1997) Self-Rated Health and Mortality: A Review of Twenty-Seven Community Studies. Journal of Health and Social Behavior 38:21–37

7.	Schnittker J, Bacak V (2014) The increasing predictive validity of self-rated health. PloS one 9:e84933

8.	VanderWeele T, Tchetgen Tchetgen EJ (2017) Mediation analysis with time varying exposures and mediators. J R Stat Soc Series B Stat Methodol 79:917–938
