# Supplementary material for: Effects of aircraft noise exposure on self-reported health through aircraft noise annoyance: Causal mediation analysis in the DEBATS longitudinal study in France
Source: PLoS One. 2024 Aug 27;19(8):e0307760. doi: 10.1371/journal.pone.0307760 (PMC11349086; doi:10.1371/journal.pone.0307760)
Supplement: Attachment — Submitted filename: Response to Reviewers.docx [file pone.0307760.s004.docx]

Responses to Reviewers’ comments

Below are the co-authors' responses to the final points raised during the review process.

**Reviewer 2 comments**

**Q3- With the term “gender” I assume you mean “sex”.**

First response of the co-authors: Not really. The gender was recorded by the interviewer without asking the participant. It may therefore differ from the biological sex.

**How did the interviewer exactly record gender?**

Response of the co-authors:

The interviewer recorded gender thanks to the following question, which was addressed to him and not to the participant: “What is the sex of the person responding to the questionnaire?”

1: Woman

2: Man”.

As the interviewer answered this question without asking the participant, there is no way of verifying whether this corresponds to their biological sex or their gender. According to the WHO definition, gender refers to “the socially constructed roles, behaviors, activities, and attributes that a given society considers appropriate for males and females”. Sex refers to “the biological and physiological factors that define males and females”^[1]^. We can assume that for the vast majority of participants, it is indeed their sex. The co-authors are therefore of the opinion that gender should be replaced by sex in the text. They have done so in the manuscript.

Q4- Do you have data on pre-existing diseases or newly developed diseases at follow-up and medication intake?

First response of the co-authors: We have information on the existence of certain pre-existing diseases such as cardiovascular disease, diabetes, cancer, diagnosed hypertension, or the use of antihypertensive medication or of other medication. Some of these have already been the subject of published articles and others will be the subject of future publications.

**I think it might be useful to control for them - do you agree?**

Response of the co-authors:

The table below shows the results of the effects of aircraft noise on self-reported health after additional adjustment for pre-existing diseases, a variable corresponding to at least one comorbidity diagnosed in the last 12 months prior to the interview, from among the following: diabetes, cardiovascular diseases, myocardial infarction, cancer, diagnosed hypertension and medication use. The results remain similar, with only a slight reduction in direct and total effects. These results have been added in S1 Table and have be commented on in the Results section.

|  | **50-54 vs < 50 dBA** | **55-59 vs < 50 dBA** | **≥ 60 vs < 50 dBA** |
| --- | --- | --- | --- |
|  | OR (95%CI^1^) | OR (95% CI^1^) | OR (95%CI^1^) |
| Total interventional effect | 1.06 (0.88 to 1.46) | 1.13 (0.99 to 1.64) | 1.20 (0.99 to 2.10) |
| Direct interventional effect | 1.01 (0.82 to 1.38) | 1.03 (0.91 to 1.40) | 1.04 (0.86 to 1.67) |
| Indirect interventional effect | 1.05 (0.96 to 1.22) | 1.10 (1.03 to 1.24) | 1.15 (1.05 to 1.39) |
| Mediated proportion^2^ | 83% | 77% | 76% |

**Reviewer 3 comments**

**The authors have addressed my comments well. However, there are still many minor grammatical errors and I recommend a close re-reading to comb through the manuscript before the submit their final version.**

An English native-speaking colleague carefully proofread the manuscript, combing through it and correcting any remaining English language errors.

1. World Health Organization (2011) Taking sex and gender into account in emerging infectious disease program: an analytical framework. WHO Regional Office for the Western Pacific.
